# *C4B *null alleles are not associated with genetic polymorphisms in the adjacent gene *CYP21A2 *in autism

**DOI:** 10.1186/1471-2350-9-1

**Published:** 2008-01-07

**Authors:** Thayne L Sweeten, Daniel W Odell, J Dennis Odell, Anthony R Torres

**Affiliations:** 1 Center for Persons with Disabilities, Utah State University, 6895 Old Main Hill, Logan, UT 84322-6895, USA; 2 Department of Biological Engineering, Utah State University, 4105 Old Main Hill, Logan, UT 84322-4105, USA

## Abstract

**Background:**

Research indicates that the etiology of autism has a strong genetic component, yet so far the search for genes that contribute to the disorder, including several whole genome scans, has led to few consistent findings. However, three studies indicate that the complement *C4B *gene null allele (i.e. the missing or nonfunctional *C4B *gene) is significantly more frequent in individuals with autism. Due to the close proximity of the *CYP21A2 *gene to the *C4B *locus (3 kb) it was decided to examine samples from autistic subjects, including many with known *C4B *null alleles for common *CYP21A2 *mutations.

**Methods:**

Samples from subjects diagnosed with autism and non-autistic controls (controls) previously typed for *C4B *null alleles were studied. Allele specific polymerase chain reaction (PCR) methods were used to determine 8 of the most common *CYP21A2 *genetic mutations, known to completely or partially inhibit 21-hydroxylase, the enzyme encoded by the *CYP21A2 *gene.

**Results:**

Although the combined autism and control study subjects had 50 *C4B *null alleles only 15 *CYP21A2 *mutations were detected in over 2250 genotypes. Eight mutations were detected in the autistic samples and 7 in the controls. The frequency of *CYP21A2 *mutations was similar between the autism and control samples. Only one individual (autistic) carried a chromosome containing both *C4B *null allele and *CYP21A2 *mutations.

## Background

Autism is a severe neurodevelopmental disorder that is approximately four times more common in males than females. The current prevalence for the disorder is approximately 1 in 152 children [1]. Although there are various mechanisms that can lead to this behaviorally defined condition, in most cases the etiology remains unknown. A strong genetic component clearly exists [2]; however, consistent detection of disease associated genetic variants has rarely been reported. Whole genome scans using microarray technology may better detect the genetic contributions to autism susceptibility [3].

Genes located in the RCCX module found on chromosome 6 in the human leukocyte antigen (HLA) locus are associated with various disease states [4,5]. This module contains the genes *RP, C4, CYP21*, and *TNX *(abbreviated RCCX) in a contiguous sequence. Different variants of these four genes can exist in the RCCX module including *RP1 *or *RP2, C4A *or *C4B *(long or short), *CYP21A2 *or *CYP21A1P*, and *TNXA *or *TNXB*. *RP2 *and *TNXA *are gene fragments while *CYP21A1P *is a pseudogene. A single chromosome usually contains one, two, three RCCX modules in tandem, but rare cases can have four modules.

**Figure 1:**
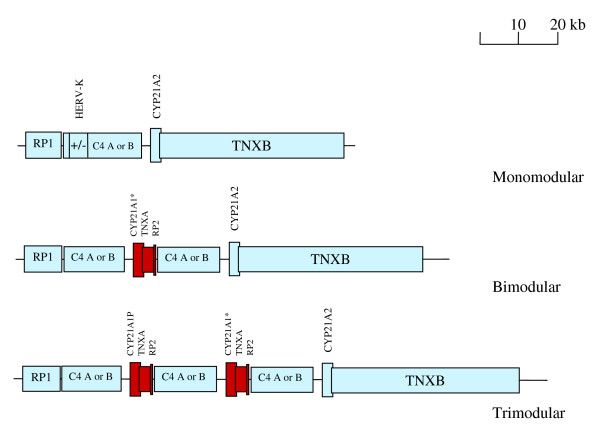
**Three common arrangements of the RCCX module**. Monomodular, bimodular, and trimodular are present in about 17, 69 and 14% of chromosomes, respectively [7]. The *C4 *gene which is either *C4A *or *C4B *can be either a long or short variant depending upon the presence of a 6.36 kb endogenous retrovirus, *HERV-K(C4)*. Pseudogenes or gene fragments are red. CYP21A1* indicates either a *CYP21A1P *pseudogene or the *CYP21A2 *gene. In the present study 40 chromosomes from autistic individuals had a *C4B *null allele. Of these chromosomes 19 were monomodular and 21 were bimodular. In the control subjects with *C4B *null alleles 10 chromosomes were monomodular and 1 was bimodular. Bimodular *C4B *null alleles were significantly more frequent in autistic subjects compared to controls (P = 0.0001). No *C4B *null alleles or *CYP21A2 *mutations were detected in the subjects (2 autistic, 4 control) with trimodular RCCX modules, determined by protein immunofixation electrophoresis [10].

Because of the diversity in the number and size of the RCCX modules misalignments and unequal crossovers occur during meiosis resulting frequently in deletions, conversions, duplications along with the acquisition of mutations from nearby pseudogenes or gene segments [6]. One such mutation in this module is the missing/nonfunctional *C4A *or *C4B *gene (*C4 *null allele). Therefore, the *C4 *containing complex is an extraordinarily complex region of the human genome [7] (Figure 1).

The RCCX module may play a significant role in the genetic underpinnings of autism. Several studies have shown that the frequency of *C4B *null alleles is increased in individuals with autism [8-10], the most recent of which found that 42.4% of autistic subjects carry a *C4B *null allele compared to 14.5% of controls [10]. The *CYP21A2 *gene is located approximately 3 kb downstream of *C4 *and the concurrent deletion of *C4B *with portions of the *CYP21A2 *has been described [11,12]. Therefore, the aim of the present research was to determine if the *C4B *null allele, found frequently in subjects with autism, is associated with *CYP21A2 *mutations. As well, the overall frequency of *CYP21A2 *mutations in autistic verses control subjects was determined.

*C4 *genes encode innate immune C4 proteins that are important in the complement cascade. *CYP21A2 *encodes an enzyme, 21-hydroxylase, which is important in the synthesis of cortisol and in maintaining proper androgen levels.

Over 2,250 genetic typings of *CYP21A2 *mutations were completed in 80 autistic and 60 controls subjects that had previously been typed for *C4B *null alleles [10]. Fifteen total *CYP21A2 *mutations where detected; however, only one individual (autistic) carried a chromosome containing both a *C4B *null allele and *CYP21A2 *mutations. Therefore, in these subjects it does not appear that *C4B *null alleles are associated with the *CYP21A2 *mutations studied.

## Methods

### Subjects

This study utilized samples previously characterize for *C4A *and *C4B *null alleles in an autism case-control study [10]. Autistic subjects and controls were Caucasian of Northern European descent and IRB (Utah State University) approval was obtained for this study. As reported, the subjects were diagnosed with autism using DSM-IV criteria by pediatric psychiatrists and psychologists expert in the evaluation of autism. The Autism Diagnostic Observation Schedule (ADOS) [13] and the Autism Diagnostic Inventory (ADI) [14] confirmed the Diagnosis. Various CYP21A2 genetic determinations were completed in 80 individuals with autism (8 female, 72 male) and 60 control subjects (15 female, 45 male). Parents of the particular subjects were typed if their child was positive for a mutation.

### DNA preparation

DNA samples were extracted from peripheral blood mononuclear cells as previously described [15]. To genotype samples with limited amounts of DNA, whole genome amplification was performed using multiple displacement amplification (MDA) based on the method of Dean *et al.*[16]. MDA was performed using RepliPHI™ Phi 29 Reagent Sets (Epicentre^®^Technologies, Madison, Wisconsin).

PicoGreen^® ^quantitation of amplified DNA was performed using a Quant-iT™ DNA Assay Kit from Molecular Probes™ (Eugene, Oregon) according to kit protocol. Fluorescence was measured with a Synergy HT microplate reader (BIO-TEK^®^, Winooski, Vermont).

### Polymerase chain reaction

Seven mutations were determined by allele-specific polymerase chain reaction (PCR) based on the method of Wilson *et al.*[17]. This method is as accurate as the dot blot procedure [17]; therefore, it is sensitive enough to detect a mutation in only one *CYP21A2 *gene if more than two copies of the gene are present. The mutations analyzed included amino acid substitutions (P30L, I172N, V281L, R356W, exon 6 cluster mutation (L236N, V237Q, M239K)), a splicing mutation (intron 2 (656) A/C to G), and a deletion (exon 3, 8 base pair deletion). Each reaction contained a primer specific for either the common or rare genetic variant in conjunction with a primer that amplified only the *CYP21A2 *gene and not the pseudogene (Table 1). A PCR based assay for detection of a 30 kb deletion/conversion affecting both *C4B *and *CYP21A2 *was performed based upon the method described in Keen-Kim *et al.*[18].

**Table 1:** Sequences of oligonucleotide primers for allele-specific PCR

**CYP21A2 Mutations [ref]**	**rs #**	**Primer**	**5'-Sequence-3'**
30 kb deletion [18]		common forward	gcttcttgatgggtgatcaat
		rare forward	tccccaatccttactttttgtc
		reverse	cctcaatcctctgcagcg
V281L [17]	rs6471	common reverse	tccactgcagccatgtgcac
		rare reverse	tccactgcagccatgtgcaa
		forward	gagggatcacatcgtcgtggagatg
I172N [17]	rs34607927	common forward	tcctcacctgcagcatcat
		rare forward	ctctcctcacctgcagcatcaa
		reverse	agctgcatctccacgatgtga
R356W [17]		common reverse	ctaagggcacaacgggccg
		rare reverse	ctaagggcacaacgggcca
		forward	gagggatcacatcgtcgtggagatg
P30L [17]		common forward	tccggagcctccacctccc
		rare forward	tccggagcctccacctcct
		reverse	agctgcatctccacgatgtga
IN2 (656) A/C to G [17]		common forward (A)	ttcccaccctccagcccccaa
		common forward (C)	ttcccaccctccagcccccac
		rare forward	ttcccaccctccagcccccag
		reverse	agctgcatctccacgatgtga
Ex 3 (8 bp deletion) [17]		common forward	cggacctgtccttgggagactac
		rare forward	actacccggacctgtccttggtc
		reverse	agctgcatctccacgatgtga
Ex 6 cluster		(see reference 17 for further description)	
L236N		common reverse	agctgcatctccacgatgtga
V237Q	rs12530380	rare reverse	tcagctgcttctcctcgttgtgg
M239K	rs6476	forward	cggacctgtccttgggagactac

### Statistics

Chi-square analysis along with the Fisher's Exact Test where performed using SPSS 14 (SPSS Inc., Chicago, Illinois). A two-tailed test with a P-value of < 0.05 was considered significant after Bonferoni corrections for multiple comparisons.

## Results and discussion

Although the 80 autistic individuals studied had 40 *C4B *null alleles and the 60 control individuals had 10 *C4B *null alleles only 15 total *CYP21A2 *mutations were detected in over 2250 genotypes. Eight *CYP21A2 *mutations were detected in the autistic subjects and 7 in the controls (Table 2). Only one individual (autistic) had a chromosome carrying both a *C4B *null allele and a *CYP21A2 *mutation. Therefore, no association was determined between *C4B *null alleles and *CYP21A2 *mutations in the study subjects.

**Table 2:** Frequencies of the *C4B *null allele and *CYP21A2 *mutations

	**Autism**	**Control**	**P-value**
	
*C4B *null allele	40/160	10/120	P = 0.0003
30 kb deletion/conversion	1/160	2/120	Not significant (NS)
			
*CYP21A2 *mutations			
V281L	3/160	1/120	NS
I172N	1/160	2/120	NS
R356W	1/160	0/120	NS
P30L	2/160	1/120	NS
IN2	0/160	0/120	NS
Ex 3 Del	0/160	1/120	NS
Ex 6 cluster	0/160	0/120	NS

Overall, the number of *CYP21A2 *mutations did not differ between the autism and control groups and no group differences were found in frequencies of individual *CYP21A2 *mutations. The frequency of *CYP21A2 *mutations seen in this research is similar to general population frequencies reported by others [19]. Five individuals with autism (6.25%) and five controls (8.33%) carried a *CYP21A2 *mutation. Two individuals with autism and one control subject carried two or three mutations. In these cases the mutations all typed to individual parents. Thus, the multiple mutations were found on single chromosomes. When a mutation was found in the child's DNA it was also present in DNA from one of the parents, thus confirming both accurate typing and absence of de novo mutations in the study children.

HLA extended haplotyes known to contain *C4B *null alleles are increased in frequency in autistic individuals (i.e. HLA extended haplotypes 35.2 (n = 4), 35.3 (n = 2), 44.1 (n = 8), and 58.1 (n = 1)). Extended haplotypes 35.3 and 58.1 have monomodular RCCX structures (*C4A*: *C4B *null allele) and extended haplotypes 35.2 and 44.1 are bimodular (*C4A*, *C4A*:*C4B *null allele). No *CYP21A2 *mutations were observed in these haplotypes.

The HLA 8.1 is present in 10% of the Caucasian population and represents the most common extended haplotype. It has a monomodular RCCX structure with a *C4A *null allele and a normal *C4B *gene (*C4A *null allele: *C4B*). This extended haplotype, referred to as COX in the MHC Haplotype Project [20], has been completely sequenced [21]. Of the eighteen subjects with an 8.1 extended haplotype one had the rare SNP creating the TTG codon that encodes for leucine at position 281 while 17 subjects had the common codon GTG that encodes for valine at position 281. This observation is in agreement with DNA sequencing data that shows low level SNP diversity in 8.1 extended haplotypes from different individuals [22].

**Figure 2:**
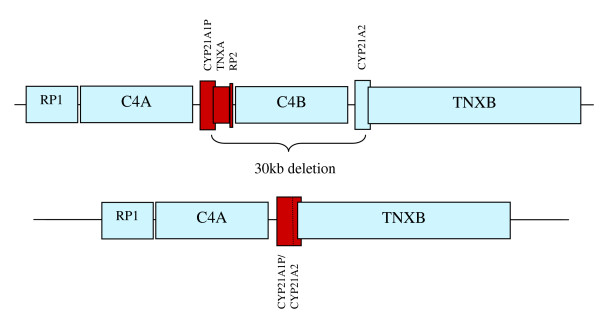
**The 30 kb deletion/conversion of *C4B *and *CYP21A2***. This diagram depicts the most common arrangement of the *RP, C4, CYP21*, and *TNX *(RCCX) gene module. Pseudogenes or gene fragments are red. The 30 kb deletion removes part of *CYP21A1P, TNXA, RP2, C4B *and part of *CYP21A2 *leaving a non-protein encoding *CYP21A1P/CYP21A2 *region.

The specific *CYP21A2 *mutations were chosen because they are the most common mutations that result in significant decreases in 21-hydroxylase activity. One of the mutations investigated was a 30 kb deletion/conversion that deletes *C4B *and converts *CYP21A2 *into a nonfunctional *CYP21A1P/CYP21A2 *hybrid with its pseudogene (Figure 2). This mutation was of particular interest because it provides a direct link between a *C4B *null allele and a *CYP21A2 *mutation. One autistic subject and two controls carried this mutation. Again, these three polymorphisms were inherited and did not involve de novo mutations.

It has been reported that two of the mutations, P30L and V281L, partially inhibit 21-hydroxylase activity (30–60% of normal), whereas the other mutations analyzed cause either complete or nearly complete enzyme inhibition [23]. Four subjects with autism (5%) carried a partially inhibiting mutation (P30L or V281L) compared to two control subjects (3.33%). This difference is not statistically significant.

21-hydroxylase deficiency is the most common cause of congenital adrenal hyperplasia. Some evidence supports the idea of 21-hydroxylase being involved in autism [24], which could result in the excessive androgen production seen in some cases [25] and thereby contribute to disease etiology [26,27]. The present data does not provide genetic support for 21-hydroxylase involvement in autism.

## Conclusion

This study examined both mono and bimodular RCCX genetic modules that contain *C4B *null alleles for mutations in the adjoining *CYP21A2 *gene. The *C4B *null alleles seen in autism are not associated with the *CYP21A2 *genetic mutations examined in this study. The frequency of *CYP21A2 *mutations was similar between the autism and control groups. Based on family typings no de novo mutations of *C4B *or *CYP21A2 *were apparent in study subjects. Therefore, the *CYP21A2 *mutations studied do not appear to contribute to the etiology of autism. However, a role for *CYP21A2 *in autism cannot be ruled out as other factors affecting *CYP21A2 *gene expression such as promoter polymorphisms or epigenetic variation were not studied and may be relevant [28]. As well, a weak association may be beyond the statistical power of the present study to detect.

## Competing interests

The author(s) declare that they have no competing interests.

## Authors' contributions

DWO aided in the molecular genetic studies. JDO assigned the C4 typing. ART aided in the study design and assays, coordination the research and helped to draft the manuscript. TLS conceived of the study, carried out the molecular genetic studies, participated in the design, performed the statistical analysis and drafted the manuscript. All authors have read and approved the final manuscript.

## Pre-publication history

The pre-publication history for this paper can be accessed here:


